# Medical students' perspectives on body donation to science within the Italian context

**DOI:** 10.1002/ase.70295

**Published:** 2026-07-13

**Authors:** Antonietta Fazio, Giulio Vara, Veronica Moretti, Annalisa Plava, Anna Maria Billi, Alessandra Ruggeri, Giulia Adalgisa Mariani, Marilisa Quaranta, Simone Lodi, Cecilia Acuti Martellucci, Maria Alessandra Sotgiu, Valentina Di Felice, Sergio Castorina, Guido Carpino, Veronica Macchi, Rita Rezzani, Mario Rende, Andrea Montella, Lucio Cocco, Lucia Manzoli, Stefano Ratti

**Affiliations:** ^1^ Cellular Signalling Laboratory, Anatomy Center, Department of Biomedical and Neuromotor Sciences (DIBINEM) University of Bologna Bologna Italy; ^2^ Department of Sociology and Business Law University of Bologna Bologna Italy; ^3^ Department of Human Sciences Link Campus University Rome Italy; ^4^ Department of Medical and Surgical Sciences University of Bologna Bologna Italy; ^5^ Department of Biomedical Sciences University of Sassari Sassari Italy; ^6^ Department of Biomedicine, Neurosciences and Advanced Diagnostic (BIND) Human Anatomy and Histology Institute, University of Palermo Palermo Italy; ^7^ Department of Medical, Surgical Sciences and Advanced Technologies “G.F. Ingrassia” University of Catania Catania Italy; ^8^ Department of Anatomical, Histological, Forensic Medicine and Orthopedics Sciences Sapienza University of Rome Rome Italy; ^9^ Department of Neurosciences, Institute of Human Anatomy University of Padua Padua Italy; ^10^ Department of Clinical and Experimental Sciences, Anatomy and Physiopathology Division University of Brescia Brescia Italy; ^11^ Department of Medicine and Surgery, Section of Human Anatomy, Clinical and Forensic Anatomy University of Perugia Perugia Italy

**Keywords:** academic survey, anatomical training, attitudes, awareness, legal framework, medical education

## Abstract

Body donation is essential for medical education and research, supporting anatomical training and clinical competence. In Italy, national data on awareness of the legislation regulating body donation and factors shaping donation‐related decisions among healthcare trainees remain limited. This study investigated knowledge of the legal framework, attitudes, and willingness for body donation among Italian medical students and residents. A two‐phase mixed‐methods study was conducted. In phase 1, qualitative interviews informed the development of a structured questionnaire. In phase 2, a national cross‐sectional survey was carried out in 2023 using a CAWI questionnaire. Items explored exposure to body‐based training, awareness of body donation and related legislation, perceived barriers and facilitators, and willingness to donate. Group comparisons and multivariable logistic regression analyses were performed. A total of 1044 participants completed the survey. Most were aware of body donation (92.8%), awareness of regulating law was limited (78.2% unaware), and public information was perceived as insufficient (69.6%). Overall, 53.8% reported willingness for body donation. Cultural and spiritual beliefs, emotional discomfort, and bureaucratic procedures emerged as major barriers. Students more often identified knowledge as a facilitator and less often viewed emotional involvement as a barrier compared with residents. In multivariable analysis, willingness to donate was negatively associated with male gender and lack of exposure or interest in body‐based training. Among Italian healthcare trainees, willingness to consider body donation is high but coexists with limited legal awareness and persistent social barriers. Educational strategies within healthcare training contexts may support informed decision‐making and greater engagement.

## INTRODUCTION

Body donation plays a critical role in medical education and research, providing essential resources for the training of healthcare professionals and the advancement of medical knowledge.^1^, ^2^, ^3^ The act of body donation offers a unique opportunity for students to engage in hands‐on learning through body dissection and prosection, both fundamental for understanding human anatomy and developing clinical competence across medical disciplines.^4^ Donated bodies are often described as the “first patients” for students and healthcare trainees,^5^ fostering not only technical training but also essential values, such as ethical responsibility and empathy. This dual benefit is essential for developing clinical expertise and professional identity in future healthcare providers.^6^, ^7^ Beyond education, body donation contributes to the advancement of medical research and supports the development of innovative surgical techniques that can directly enhance healthcare outcomes.^8^

Although emerging technologies such as augmented or virtual reality have expanded the range of tools available for anatomical teaching,^9^, ^10^ they cannot replace the pedagogical and professional value of learning with donated bodies, which remains essential for achieving clinical competence.^11^ Ensuring access to this irreplaceable learning experience thus requires robust and sustainable body donation programs that provide adequate availability of donated human bodies for medical training.

Globally, body donation programs exhibit considerable heterogeneity in both structure and effectiveness. In countries such as the United States, Australia, and New Zealand, long‐standing and well‐established body donation programs reliably provide academic institutions with sufficient donors for teaching, clinical training, and research.^12^, ^13^ These programs operate with strong ethical oversight and formal recognition of donors, incorporating pre‐death registration and next‐of‐kin consent to ensure informed consent, procedural transparency, and commemoration of donors.^14^ By contrast, many countries still rely primarily on unclaimed bodies rather than voluntary donations due to cultural and religious barriers, limited public awareness, or inadequate regulatory frameworks.^15^, ^16^, ^17^ Although the International Federation of Associations of Anatomists (IFAA) recommends the exclusive use of willingly donated bodies, unclaimed bodies continue to be used in several regions.^16^

Recent legal reforms, including those introduced in Italy, aim to strengthen regulatory clarity and promote greater awareness of body donation, moving the system closer to international ethical standards. Specifically, Law No. 10/2020 established a structured framework for the post‐mortem donation of bodies and tissues for educational and research purposes by designating specific reference centers.^18^ It also specifies the duration of donation, management of corpses, and compatibility with organ donation. Despite these advances, operational limitations and ambiguities remain in the implementation phase^18^, ^19^ and awareness of the law is still limited, even among students and faculty.^20^, ^21^ This gap highlights the need for targeted educational initiatives to improve understanding and participation in body donation programs.

Increasing awareness and motivation among healthcare professionals is relevant. Studies indicate a persistent discrepancy between their professional support for body donation and their lower personal willingness to participate as donors.^22^, ^23^ Of note, medical students as well as residents are the primary beneficiaries of body donation programs, positioning them to effectively communicate the educational and societal value of donation. As future physicians, they can act as intermediaries between the healthcare system and the public, shaping perceptions and disseminating accurate information about body donation. Their ability to share personal reflections and evidence‐based information, grounded in firsthand experience, may positively influence public attitudes toward donation. This intermediary role is recognized in medical literature as essential for sustaining ethically robust and culturally sensitive body donation programs.^24^

However, willingness to donate is not determined solely by educational exposure. It is shaped by a complex interplay of cultural, ethical, and personal factors that remain only partially understood.^20^ These determinants can strongly influence awareness, attitudes, and willingness to donate, even among healthcare professionals. Reported concerns include discomfort with the idea of their body being dissected, familial opposition, and cultural taboos,^25^ while altruism frequently emerges as a powerful motivational force,^26^ reflecting gratitude and the desire to contribute to future generations.^27^, ^28^ Clarifying how these attitudes evolve during training is crucial for designing effective educational strategies that may support efforts to improve body donation programs in Italy.

Building on this premise, the present study examines the knowledge, attitudes, and willingness of future and practicing Italian healthcare professionals to donate their own post‐mortem bodies, with the aim of elucidating the key concerns, limitations, and motivations behind this complex decision. By analyzing the intricate interplay of cultural, ethical, and personal factors, this work seeks to inform strategies to promote body donation within medical education and healthcare training contexts in Italy.

## MATERIALS AND METHODS

### Ethical considerations

The present study received approval by University of Bologna Bioethical Board (Prot. N. 0394338 del 20/12/2022). Informed consent was obtained from all participants involved in both the qualitative and quantitative phases of the study. For the interview phase, participants provided written informed consent prior to participation and to audio recording. For the survey phase, completion and submission of the questionnaire were taken to indicate consent. No incentives, monetary or otherwise, were provided, ensuring that participation remained entirely voluntary and free from potential bias. The questionnaire was administered in an anonymous form, and no personal identifiers were collected.

### Study design and participants

This study adopted a two‐phase mixed‐methods design, integrating an exploratory qualitative phase which informed the development of a subsequent survey. The overall aim was to develop and validate a questionnaire to assess knowledge, attitudes, and willingness toward post‐mortem body donation among medical students and residents. To guide this exploration, the following research questions were formulated:

1. What are the attitudes and opinions of medical students and residents regarding the donation of one's body to science post‐mortem?
2. How does the level of anatomical experience and professional background influence the willingness of students and residents to consider body donation for scientific purposes?

### Phase 1: Exploratory qualitative study

This preliminary phase, carried out from October 2022 to May 2023, employed a qualitative method to gather a nuanced understanding of the social and personal dimensions associated with body donation. Unstructured interviews were conducted which were audio‐recorded with participant consent and transcribed verbatim. Thematic analysis was subsequently conducted using an inductive approach. The use of unstructured interviews enabled an exploratory and inductive engagement with participants' narratives, allowing culturally embedded meanings and experiential dimensions to emerge without being constrained by predefined categories.

Participants for interviews were recruited from senior and junior medical students, and healthcare professionals affiliated with the University of Bologna (Italy), as well as living donors and family members from the Emilia‐Romagna Region. In addition, experts in medical ethics and sociology were included. Participants were selected using purposive sampling to ensure a diverse and information‐rich group based on profession, experience with body dissection, and relationship to body donation. Individuals were approached via academic networks, institutional contacts, and direct invitations within the medical education context. Inclusion criteria involved being a medical student (junior or senior), resident, and healthcare professionals. Participants had to be over 18 and willing to discuss their experiences and views on body donation.

### Data analysis

Transcripts were imported into Nvivo12 software (QSR International, 2018) for coding and analysis. Initial codes were independently generated by two researchers (V.M. and A.P.) and subsequently discussed to ensure consistency and resolve discrepancies. These interviews explored the meanings individuals ascribed to body donation, allowing culturally embedded interpretations and experiential dimensions to emerge. Findings were synthesized into a thematic map, which visually provides the relationship, dynamics, and common concepts among participants.

### Study outcomes

Seventy‐five interviews were conducted, with an average duration of approximately 45 min. Thematic saturation was reached when further data collection produced no additional insights relevant to the analytical categories (see cognitive maps in Table 1), thus providing a comprehensive social, cultural, and technical framework of body donation. Themes emerging from the qualitative phase, grounded in participants' own language, included modesty (concerns related to dignity, privacy, and the perception of the body as “sacred,” particularly among relatives), emotional participation (ranging from initial discomfort to gratitude and a sense of responsibility, especially among students and professionals), bureaucracy (described as manageable when mediated by specialized centers but challenging when involving local health authorities and administrative procedures), knowledge/consciousness (often shaped by informal sources such as media or personal networks, and uneven even among healthcare professionals), and end‐of‐life interpretation (frequently framed in terms of altruism, legacy, and the idea of the body as a “useful” entity after death). Importantly, these themes varied across participant groups. For instance, surgeons emphasized the ethical and educational value of donation for patient safety, whereas relatives highlighted the emotional and relational burden of decision‐making, and students described a process of gradual familiarization balancing respect, curiosity, and professional growth. Although framed in open‐ended terms, these categories were deliberately retained in the survey to capture how participants positioned themselves toward culturally and emotionally charged concepts. These results directly contributed to the creation of the quantitative instrument through the following steps: (1) identification of survey variables (items) by translating the main categories into specific questions or rating scales; (2) the formulation of items using participants' own language to enhance clarity and cultural relevance for the target audience; (3) the construction of closed response options reflecting lived experiences; (4) operational difficulties and limited awareness of body donation practice and legislation informed the section addressing bureaucratic processes.

**TABLE 1:** Cognitive maps derived from the qualitative phase, illustrating thematic categories and sub‐themes across different participant groups, including (A) donors' relatives, (B) body donors, (C) surgeons, (D) students and tutors, and (E) their cross‐cutting future perspectives.

(A) Donors relatives	
Main category	Sub‐themes and details
Profile and family experience	*Relationship with the body*: Seen as a utility; no sacred importance *Experience*: The choice falls on those “left behind”; mentally demanding; “desire/wait” for the return; gradual detachment *Spirituality & Death*: Death as a “passage”; no taboos; non‐obstructive spirituality
Encounter with donation	*Learning/Discovery*: Through TV, radio, exhibitions (e.g., Body Worlds), articles, word of mouth, or family precedents (France/Switzerland) *Motivations*: History of illness in the family; desire to be useful; gratitude for medical care received; belief in scientific progress and the community
Meaning of the gesture	*Values*: Altruism, generosity, pride, love *Vision*: “Prolonged life”; giving meaning to illness; consistency and concrete value for research
Social reactions	*Close Relatives/People*: Wonder, pride, stimulus to follow the same path (though sometimes with initial fear or lack of sharing) *Other relatives/Friends*: Ranges from total closure and lack of understanding to moral support and shared acceptance *External*: Curiosity, amazement, sometimes criticism or lack of interest, but generally well‐disposed
The process (path)	*Bureaucratic activation*: Simple when done through the Center; difficult when coordinating with Municipalities, Health Authorities (ASL), or GPs/hospices
Resistance and barriers	*Obstacles*: Strong religious beliefs; the idea of the “sacred” body and modesty; psychological commitment; delaying funeral rites and related timing
Awareness strategies	*Actions*: Storytelling; digital/TV information; engaging schools and universities; discussing “green” burial forms; using respected public figures for outreach

(B) The body donor	
Main category	Sub‐themes and details
Relationship with the body and spirituality	*Body*: Viewed as a “chrysalis” that evolves after death, a shell with no importance, or pure utility *Death/Spirituality*: Absence of taboos or obstructive beliefs; death is perceived as a “passage,” a natural and “just” event
Encounter with donation	*Learning/Discovery*: Occurs through the internet, TV, friends, international cases, or even the donation of a pet's body *Motivations*: Desire to be useful for science and future generations; ethical choice; sense of community; awareness of “passing away”
Meaning of the gesture	*Personal perception*: Considered as “the last good thing to do in life”; a contribution to research; a right action that “transmits” something; sometimes with a touch of narcissism
Communication and social reactions	*Close relatives/People*: Natural dialogue and a choice often shared without conflict, despite initial cultural hesitations *Relatives/Friends*: Mixed reactions ranging from closure and difficulty understanding the choice to being perceived as “eccentric” or “contrarians” *External*: Often marked by lack of awareness, lack of knowledge, and amazement even within primary care medicine
Operational aspects and strategies	*Process*: Simple if managed with the Center; more difficult to implement with Municipalities, Health Authorities (ASL), and family doctors (especially post‐2020 law) *Awareness*: Word of mouth, school involvement, commercials about “progress,” and distribution of materials in sensitive facilities
Resistance	*Barriers*: Idea of the body as “sacred”; modesty; reluctance toward cremation; death taboos and limits related to religiosity

(C) Surgeons	
Main category	Sub‐themes and details
The surgeon's role today	*Skills*: The need to navigate complex pathological anatomies; the importance of direct clinical experience *Relational aspect*: Cultivating human contact; past experience projects onto and informs current practice
Training with bodies	*Safety*: A surgeon without dissection practice is considered “extremely dangerous” *Learning*: Deep knowledge of anatomy and a conscious approach to problems; calmness in the surgical gesture and new stimuli
Vision of the anatomy center	*Value*: Seen as a “Unicum” and a “Geographic Atlas” for surgical navigation; enables virtuous teaching without the need to go abroad
Relationship with the body	*Respect*: Practice of surgical suturing after dissection as a sign of respect; the scientific aspect prevails over the emotional one *Ethics*: Rigorous ethical preparation; a way to approach the “other” and value the donor's generosity
Meaning of the act of giving	*Ethical value*: A fundamental act with infinite benefit; absolute gratitude toward those who, in death, come to the aid of life *Involvement*: A strong ethical choice that leads some doctors to state: “I have decided to donate myself”
Resistance and barriers	*Cultural*: The idea of the “violated” body; “splatter” cinematic/literary narratives; the historical tendency of societies to bury the deceased *Goal*: Creating a new culture and “forma mentis” to increase awareness
**Involved Specializations** The map highlights that donation is crucial for several medical branches: Neurosurgery General Surgery Otolaryngology (ENT) Hematology Orthopedics	

(D) Students (junior and senior tutor)	
Main category	Sub‐themes and details
Role of the tutor	*Characteristics*: A dynamic role of continuous growth and rediscovery of organs and structures *Approach*: Changes the way of studying; activity aimed at medical students, but also at experts and outsiders
Relationship with students	*Context*: Peer‐to‐peer dialogue in a friendly, stimulating, and passionate environment *Guidance*: The tutor serves as a point of reference with a sense of responsibility *Group dynamics*: Based on trust and the feeling of being “in the same boat” through continuous side‐by‐side support
Training experience with bodies	*Method*: A gradual approach; it is the first opportunity to set aside books for practical work *Learning*: Learning to “think about what is inside the patient” for educational and research purposes *Distinction*: It is emphasized that an autopsy is different from a dissection
Relationship with the deceased body	*Feelings*: Gratitude, respect, curiosity, and serenity within a complex context *Awareness*: Learning the value of the donated body while guiding someone else's learning
Meaning of the act of giving	*Value of the act*: Defined as a generous, altruistic, free act of extreme trust *Political vision*: Understood as an act aimed at normalizing donation *Metaphor*: Described as a “rainbow of colors” compared to the “non‐color” of those who do not donate
The coordinator	*Recognition*: Receives the highest symbolic recognition (ringing the bell in the dissecting room, a ritual passed down like a change of government)
**Key Points of the Experience** **Balance:** The experience is described as “balancing on a rope” in a journey that brings advantages in both directions (giving back and receiving). **Purpose:** The post‐mortem body concretely helps those performing a role for those who are alive or for those who have passed. **Communication:** The need to use different languages to communicate, organize, and reassure regarding a gesture that requires it.	

(E) Future			
Perspective	Key themes and meanings	Operational and relational aspects	Resistances and challenges
The donor	Ethical and altruistic act; a gift for future generations; the body is viewed as a “chrysalis” or a useful shell	Choice often shared with “close” family members; complex activation involving Municipalities and Local Health Authorities (ASL)	Idea of the “sacred” body; death taboos; lack of information within primary care medicine
The surgeons	Absolute gratitude; fundamental act for patient safety; “coming to the aid of life”	Indispensable practical training (dissection as a “key point”); respect for the body through surgical suturing	Cultural prejudices regarding the “violated” body; “splatter” narratives; the need to create a new “forma mentis”
Tutors and students	Act of extreme trust and generosity; a “rainbow of colors” vs. the “non‐color” of non‐donation	Peer‐to‐peer dialogue between tutors and students; gradual learning and responsibility; distinction between autopsy and dissection	Difficulty in managing different languages to communicate and reassure regarding the act
Future vision	Integration between the real body and Artificial Intelligence; the body as a source of macroscopic and molecular data	Transversality between public and private sectors and between different countries; creation of advanced surgical simulations (vascularization/ventilation)	Management of the new post‐2020 law; the need to systematize donation in a more rigorous manner
**Synthesis of Cross‐cutting Concepts** **Value of the Act:** For all figures involved, donation is not just a scientific act but a gesture of profound ethical and social value, representing a “giving back” to the community. **Training:** The human body remains an irreplaceable anatomical model, even in the face of technological advancement, essential for clinical safety and professional growth. **Evolution:** There is a visible transition from a phase of “unawareness” and bureaucratic difficulties toward a future of greater systematization and technological integration.			

### Phase 2: Questionnaire development and survey

Following interviews and thematic analysis, a structured questionnaire was developed to systematically capture attitudes, perceptions, and motivations toward post‐mortem body donation among healthcare‐related participants in Italy (Supplementary Material S1). The questionnaire targeted two specific groups: (A) students enrolled in the School of Medicine and Surgery and the School of Dentistry, and (B) medical residents from all specialties. This design enabled a comprehensive understanding across varying levels of experience and roles.

The questionnaire was designed to investigate four core areas relevant to perceptions and attitudes toward body donation. First, professional training and anatomical education were explored, focusing on participants' exposure to body dissection and other forms of practical anatomy training. Second, knowledge and perception of body donation were assessed, including awareness of post‐mortem body donation for scientific purposes and familiarity with national legislation (Italian law No. 10/2020). Third, perceived barriers and motivations related to body donation were examined. Finally, strategies for public awareness and engagement were evaluated, with participants asked to assess predefined initiatives aimed at enhancing public awareness and attitude toward body donation. It is important to note that all relevant information regarding body donation was provided to ensure accurate and meaningful answers. Overall, the final questionnaire included 21 items distributed across these four thematic sections, as reported in Supplementary Material S1.

Before distribution, the questionnaire underwent a pre‐test with a sample group sharing the same characteristics as the target population. This pre‐test collected feedback on the clarity and comprehensibility of the items, allowing us to refine the wording and structure of the questionnaire to ensure accessibility, relevance, and alignment with the study's objectives. In a subsequent phase of the project, a process of participant feedback on the study findings (often referred to as member checking) was conducted. Participants were invited to review and comment on the coherence between the aggregated results and their own experiences. This process did not aim to “validate” participants, but rather to assess the interpretative consistency and sociological plausibility of the findings.

### Questionnaire distribution

The questionnaire was distributed nationwide within Italy using the CAWI (Computer‐Assisted Web Interviewing) method from June to November 2023, ensuring wide accessibility and reach among the target populations. To maximize participation rates and ensure a large and representative sample, key stakeholders in medical education were actively involved. The Intercollegiate President of the Medical Area facilitated widespread dissemination at the national level. Within the University of Bologna, the Heads of the Departments of the School of Medicine and Surgery and School of Dentistry, as well as of the Medical Specialization Schools, promoted local participation. Additionally, all faculty members in the field of human anatomy were contacted through official communications from the board of the College of Anatomy Professors of the Italian Society of Anatomy and Histology (SIAI) to support the dissemination of the questionnaire. These stakeholders were not part of the research team, and their role was limited to dissemination activities; they were not involved in data collection or analysis. To further enhance engagement, two reminder calls were carried out during the questionnaire distribution period—one three months after it started and another shortly before the deadline.

### Statistical analysis

The results of the questionnaire were expressed as means ± standard deviations (SD) for the questions with Likert scale responses, or proportions for the questions with categorical answers. The Kolmogorov–Smirnov test was used to assess the normality of the data distributions (*p* < 0.05). Different item formats were used in the questionnaire. The perceived educational value of training on donated bodies was assessed using a 5‐point Likert scale (1 = strongly disagree; 5 = strongly agree), while attitudinal statements related to body donation were rated on a 4‐point Likert scale (1 = strongly disagree; 4 = strongly agree), to encourage a directional response. For items exploring factors influencing willingness to donate, participants were asked to indicate whether each factor was perceived as an obstacle, a favorable element, or irrelevant.

Differences by age‐class, gender, and other students' characteristics were assessed according to the type of variable and comparison. For two‐group comparisons, mean Likert scores were compared using Student's *t*‐tests, which were considered appropriate given the large sample size and their robustness to moderate departures from normality, whereas proportions of positive responses were compared using chi‐squared tests. For comparisons across more than two groups, Likert‐type responses were analyzed using a Kruskal–Wallis test as a conservative nonparametric approach for ordinal data; when the overall test was significant, post hoc pairwise comparisons were performed using the Mann–Whitney *U* test with Bonferroni correction for multiple testing. Natural language analysis on the open‐ended responses on reasons for considering or not considering body donation was performed by grouping free‐text motivations according to response categories (“Yes,” “No,” “I don't know,” “I prefer not to answer”) and extracting the 20 most frequent content words (stop‐words removed).

Multivariable logistic regression analyses were used to identify independent predictors of two categorical outcomes: agreement with body donation as a way of interpreting the end of life, and considering being a body donor in the future. The regression models were defined a priori including potential confounders (age and gender) and other responses to key questions. Each covariate was tested for multicollinearity, for potential interaction and/or quadratic/cubic terms. Finally, the outlier analysis was based upon the calculation of Pearson, standardized and studentized residuals, and also Cook's D influence. We found 23 influential observations, and we repeated the analysis excluding these observations, with no substantial changes.

Variables related to institutional setting (University of Bologna) and level of training (students versus residents) were not systematically applied across all analyses. They were entered selectively when conceptually relevant to the outcome of interest. This approach was adopted to capture meaningful differences without introducing redundancy or over‐adjustment.

The results of the logistic analysis are presented as odds ratio (OR:) and 95% confidence interval (CI:). Statistical significance was defined as a two‐sided *p*‐value < 0.05 for all analyses, which were performed using Stata 13.1 (Stata Corp., College Station, TX). Exploratory psychometric validation was conducted on the responses. Sampling adequacy was evaluated with Kaiser–Meyer–Olkin test (KMO), factor analysis, Bartlett's test, and Cronbach's α. Internal consistency of the four‐item attitude scale was evaluated after reverse‐coding the negatively‐worded item. Finally, a Large Language Model (ChatGPT‐4o) was used to support the qualitative analysis of open‐ended responses due to its ability to efficiently process large volumes of text and identify recurring themes in a systematic and reproducible manner. The model was employed to assist in the initial coding and thematic grouping of responses, while the final interpretation and validation of themes were performed by the authors. Moreover, ChatGPT‐4o was also used to assist with language editing.

Seventeen questionnaires were excluded due to incomplete responses; given the relatively low percentage (~2%), no missing data imputation technique was adopted.

## RESULTS

### Demographics

A total of 1044 participants completed the questionnaire, including 849 students and 195 residents. The study population was characterized by a higher representation of female participants compared with males, with a small number of respondents identifying as other. The mean age of the sample was 24.5 years, consistent with the expected age range of individuals undergoing medical education and postgraduate clinical training. Most participants were affiliated with universities located in the Emilia‐Romagna region, while a minority were enrolled in other Italian regions. With respect to academic background, most respondents were enrolled in or had completed a degree program in Medicine and Surgery, followed by Dentistry, with a smaller proportion reporting other or unspecified degree programs. The demographic characteristics of the study population are presented in Table 2. Unless otherwise specified, results are presented for the overall sample. Comparisons between medical students and residents are reported separately in the *Group Analysis section* to explore differences according to training stage.

**TABLE 2:** Demographic characteristics of the study population (*n* = 1044), including age (mean ± SD), gender distribution, academic background, and location and level of training, expressed as frequencies and percentages.

Category	Percentages (%)	*N*	Mean (SD)
Total participants	100	1044	
Students	81.3	849	
Residents	18.7	195	
Gender			
Male	38.4	401	
Female	59.7	623	
Other	1.9	20	
Age			24.5 (±2.4)
University location			
Emilia Romagna	82.6	862	
Rest of Italy	17.4	182	
Degree program			
School of Medicine and Surgery	84.2	880	
School of Dentistry	8.1	85	
Other/unspecified	7.7	79	

### Previous experiences

Regarding training on donated bodies, a minority of participants attended faculties which do not currently implement this activity in the study course (15.2%; *n* = 159) (Table 3A). Among those with access to body‐based training, observational activities through prosections were the most common, whereas a smaller proportion participated in hands‐on training activities involving both observation and dissection of donated bodies (62.5%; *n* = 652 and 17%; *n* = 177, respectively). Training activities were predominantly held on both whole bodies and separate segments (84.9%; *n* = 704), whereas a minority involved only separate segments (15.1%; *n* = 125).

**TABLE 3:** Previous educational experiences on donated bodies. (A) Availability of training activities involving observation and/or dissection of donated bodies within the participant's institution (%). (B) Participation in courses using donated bodies at institutions other than the participant's own, including willingness to attend such courses.

(A)		
Statement	Yes (%)	No/Don't know (%)
In the course of study/faculty where you study/work, are there training activities that involve observation and/or dissection of donated bodies?	84.8	15.2

(B)			
Statement	Yes (%)	No, but I would like to attend a course (%)	No, and I would not like to attend a course (%)
Have you ever attended courses using donated bodies in institutions different that the one in which you study/work?	21.7	73.2	5.1

Among all participants, most had never attended an extra‐curricular course involving donated bodies (73.2%; *n* = 764), while approximately one quarter reported previous participation, either through personal expenses or via university conventions or scholarships (21.7%; *n* = 226). Few participants were not interested in taking part in this activity (Table 3B).

### Awareness and sources of information

Most of the participants were aware of the possibility of donating the body to scientific research (92.8%; *n* = 969). The course of study attended was the primary source of information (53.8%; *n* = 562), followed by social media (14.8%; *n* = 155) and relatives or friends (10.5%; *n* = 110). The remaining sources included colleagues, scientific literature, and academic events.

Despite this high level of awareness, respondents largely perceived public knowledge of body donation as limited, with the majority of participants reporting insufficient information (69.6%; *n* = 726) and nearly one fifth indicating that the topic was entirely unknown (19.5%; *n* = 204). Therefore, the need for dedicated information campaigns was strongly endorsed (90%; *n* = 950).

Awareness of the Italian law regulating body donation was limited, with most participants reporting no prior knowledge of the law (78.2%; *n* = 816). Moreover, opinions regarding the compatibility between body and organ donation were heterogeneous: while a majority of participants considered it possible (64%; *n* = 668), a substantial proportion remained uncertain or did not consider the two practices compatible (24.8%; *n* = 259 and 11.2%; *n* = 11, respectively).

Strategies to improve awareness of body donation were explored by asking participants to select among predefined options. The most frequently selected strategies included sharing formative experiences of students and professionals (26.34%; *n* = 275), sharing stories of donors and their families (25.00%; *n* = 261), followed by initiatives delivered through schools and universities (15.23%; *n* = 159), and through media and social media channels (13.31%; *n* = 139). Less frequently selected options included dissemination through specialized socio‐health structures (7.95%; *n* = 83), information provided by family doctors (6.61%; *n* = 69), and the creation of specific associations (4.41%; *n* = 46). Only a small number of participants chose the “Other” category; most comments did not propose alternative strategies but instead indicated that “*all previously listed options*” were considered relevant (1.06%; *n* = 11).

### Perceived educational value of body donation

A strong consensus emerged regarding the educational value of training on donated bodies, with the large majority of participants agreeing that “*Training on donated bodies is essential for gaining a deep understanding of anatomy and addressing potential critical situations/emergencies in the healthcare field more effectively*” (Likert scale: 5 = 73.1%; 4 = 20.5%).

Participants were then asked to express their level of agreement with a set of additional statements using a 4‐point Likert scale. Consistently, most participants considered body donation as “*an act of altruism/solidarity*” and “*useful to healthcare progression through training and research*” with a high level of agreement. By contrast, opinions were more divided when body donation was framed as “*a way of interpreting the end‐of‐life*.” The vast majority of participants rejected the idea that “*body donation is an inappropriate act*.” Detailed distributions for all items are reported in Table 4.

**TABLE 4:** Opinions on body donation utility and meaning. Responses to attitudinal statements were measured using a 4‐point Likert scale (strongly disagree to strongly agree) and are reported as percentages (%).

Statement	Strongly disagree (%)	Disagree (%)	Agree (%)	Strongly agree (%)
Body donation is an act of altruism/solidarity	1.3	16.5	31.9	50.3
Body donation is useful to healthcare progression through training and research	1.0	6.1	22.5	70.4
Body donation is a way of interpreting the end of life	10.0	39.5	26.0	24.5
Body donation is an inappropriate act	77.7	20.9	0.6	0.8

### Willingness to donate and underlying motivations

Participants were asked “*Would you consider becoming a body donor in the future?*” in order to assess whether such a decision could be envisaged during one's lifetime. Just over half of the participants expressed a positive attitude (53.8%; *n* = 562), while a substantial proportion reported uncertainty (33.2%; *n* = 347). Smaller proportions indicated that they would not consider donation (6.4%; *n* = 67) or preferred not to answer (6.6%; *n* = 68). Affirmative answers were primarily motivated by altruistic and scientific considerations (e.g., *My body can be useful to future generations to learn and improve patient care*), whereas negative responses reflected concerns related to body integrity (e.g., *I cannot imagine my body being subjected to dissection*) and emotional discomfort (e.g., *The thought of death frightens me greatly*). Uncertain responses were mainly characterized by information needs (e.g., *I do not know whether donation to science is compatible with organ and tissue donation*) and reliance on family consensus (e.g., *I would need my family's agreement*). Decisional uncertainty and emotional considerations predominated among participants who preferred not to answer (e.g., *To date, I have never seriously thought about it*). Results are summarized in Table 5.

**TABLE 5:** Summary of open‐ended responses on body donation, showing the most frequent themes and corresponding percentages (%) across response groups.

Group	Top theme(s) and %
Yes	Altruism/Social utility (38%), Research/Scientific progress (33%), Future generations/Legacy (18%)
No	Body Integrity/Burial (42%), Fear/Loathing (27%), Religious/Spiritual reasons (19%)
I don't know	Need for more information/Doubts (31%), Family decisions/Consent (24%), Altruism (12%)
I prefer not to answer	Decisional uncertainty/Unease to respond (>50%)

### Factors influencing attitudes toward body donation

Beyond individual motivations, participants were asked to evaluate the role of several factors in shaping attitudes toward body donation. Religion and spirituality, cultural traditions, and bodily modesty emerged as the most frequently perceived barriers. Bureaucratic procedures were also commonly identified as an obstacle. By contrast, emotional participation and the thought of death were associated with more heterogeneous responses, being viewed both as barriers and as potential facilitators. Instead, knowledge and awareness were predominantly considered as factors facilitating body donation (Table 6).

**TABLE 6:** Opinions on the factors potentially influencing body donation, categorized as obstacle, not relevant, or facilitating factor, reported as percentages (%).

Factor	Obstacle (%)	Not relevant (%)	Predisposition (%)
Religion/spirituality	86.8	9.9	3.3
Thought of death	48.2	28.6	23.2
Sense of modesty for one's body	76.8	17.0	6.2
Emotional participation	55.9	12.3	31.8
Bureaucracy	65.9	26.0	8.1
Knowledge and consciousness	15.3	6.6	78.1
Cultural traditions	80.2	12.5	7.3

### Group analysis

Significant differences were found between students and residents regarding factors influencing the decision to donate the body (Figure 1). Knowledge and consciousness were generally considered favoring factors, with a significantly greater impact among students compared to residents (79.1% vs. 68.9%, respectively, both *p* < 0.01). Students also valued emotional participation as significantly less of an obstacle compared to residents (54.8% vs. 63.9%, p < 0.01). A similar trend was observed for cultural traditions, which were regarded as an obstacle by 87.7% of residents, compared to 79.3% of students (*p* = 0.02). No significant differences were identified for the remaining factors.

**FIGURE 1:**
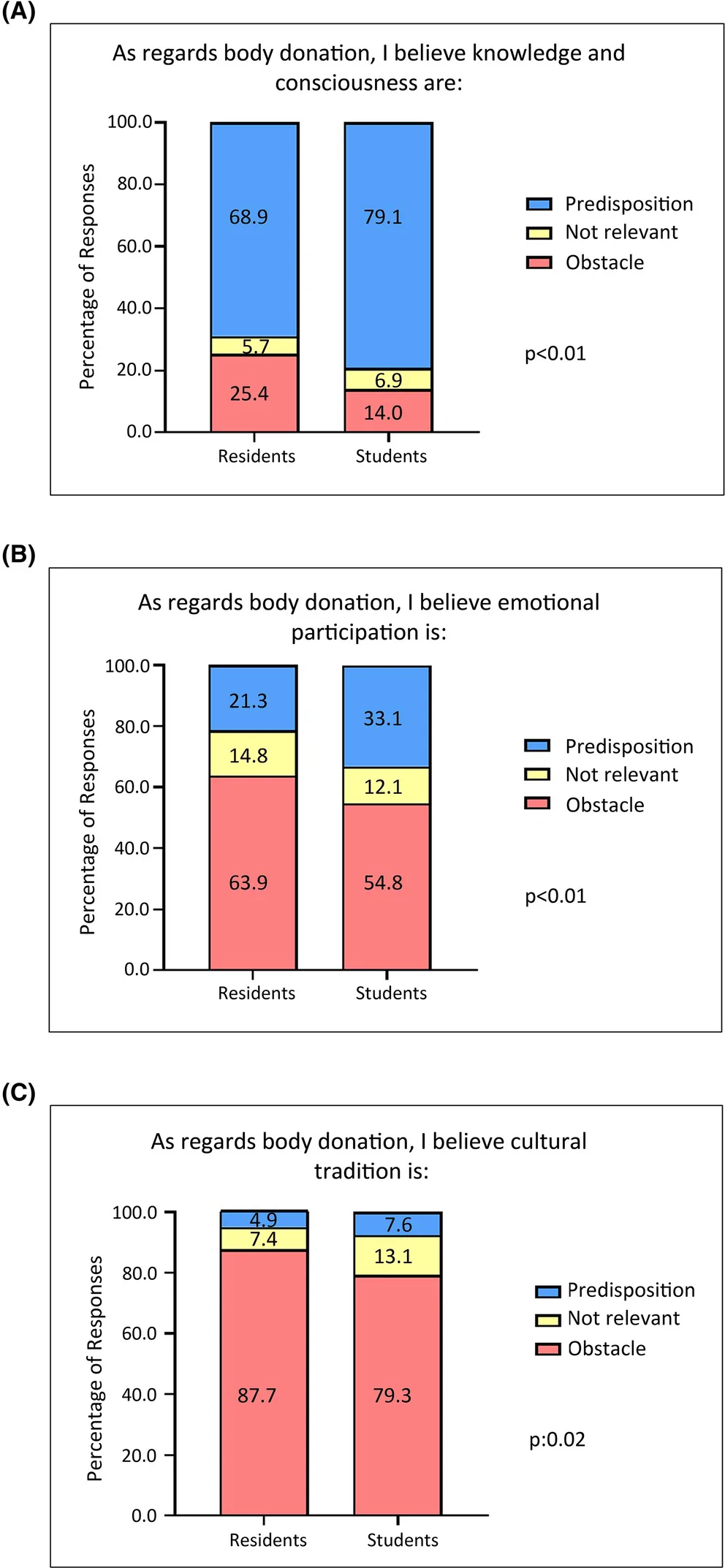
Perceived influence of selected personal factors on post‐mortem body donation among students and residents. Participants classified (A) knowledge and awareness, (B) emotional involvement and (C) cultural tradition as either obstacles, predisposing factors toward body donation, or not relevant to their decision‐making. Percentages represent the distribution of responses between residents and students. *p* values are reported in each panel.

### The experience of university of Frankfurt am Main:Bologna

Students from the University of Bologna were significantly more involved in training activities using donated bodies (Fisher's exact test, *p* < 0.001) and were more aware of the possibility of body donation compared with students from other institutions (Fisher's exact test, *p* < 0.001). At the same time, they were more likely to perceive bureaucratic procedures as an obstacle to donation (Fisher's exact test, *p* = 0.013). Students from the University of Bologna also more often endorsed body donation as a way of interpreting the end of life, with an average difference of 0.22 on the Likert scale (Mann–Whitney *U* test, *p* = 0.031).

### Multivariable analyses

In multivariable analysis, agreement with the statement “*body donation is one way of interpreting the end of life*” was independent of gender, but was more frequent among older participants (≥ 24 years). A strong positive association was observed with considering body donation an act of solidarity (OR: 4.85; 95% CI: 3.26–7.21), while previous exposure to body dissection and having considered body donation personally were also positively associated. None of the other responses predicted disagreement with this statement (Table 7).

**TABLE 7:** Results from the multivariable analysis evaluating the potential predictors of the belief the body donation is one way of interpreting the end of life.^a^

I agree that body donation is one way of interpreting the end of life^a^	% (*n*/*N*)	*p* ^c^	OR: (95% CI)	*p* ^d^
Overall sample	50.5 (527/1044)	—	—	—
Age in years				
<24	45.8 (292/637)		1 (Ref. cat.)	—
≥24	57.7 (235/407)	<0.001	1.73 (1.31–2.30)	<0.001
Gender				
Female	49.8 (305/613)		1 (Ref. cat.)	—
Male	51.7 (222/431)	0.581	1.06 (0.81–1.39)	0.682
In the course of study/faculty where you study/work				
No training activities are organized that involve neither observation or dissection of donated bodies/ Don't know	40.6 (87/214)		1 (Ref. cat.)	—
Training activities are organized that involve observation and/or dissection of donated bodies	53.0 (440/830)	0.001	1.82 (1.30–2.55)	<0.001
Have you ever attended courses using donated bodies in institutions different than the one in which you study/work?				
Yes	55.5 (126)		1 (Ref. cat.)	—
No, but I would like to attend a course	49.6 (379)		0.81 (0.58–1.13)	0.214
No, and I would not like to attend a course	41.5 (22)	0.120	0.76 (0.39–1.47)	0.417
Body donation is an act of altruism/solidarity				
No	20.4 (38)		1 (Ref. cat.)	—
Yes	57.0 (489)	<0.001	4.85 (3.26–7.21)	<0.001
I consider being a body donor in the future				
No/Not known	41.4 (200)		1 (Ref. cat.)	—
Yes	58.2 (327)	<0.001	1.72 (1.30–2.27)	<0.001
As regards body donation, I believe religion/spirituality is^b^				
An obstacle	50.8 (451)		1 (Ref. cat.)	—
Helpful	38.2 (13)		0.61 (0.28–1.30)	0.197
Not relevant	49.5 (50)	0.352	1.08 (0.68–1.70)	0.752
As regards body donation, I believe a sense of modesty for your body is^b^				
An obstacle	49.9 (392)		1 (Ref. cat.)	—
Helpful	50.8 (32)		1.08 (0.60–1.95)	0.797
Not relevant	51.7 (90)	0.903	0.99 (0.69–1.43)	0.967
As regards body donation, I believe emotional participation is^b^				
An obstacle	50.0 (286)		1 (Ref. cat.)	—
Helpful	49.9 (162)		0.93 (0.69–1.26)	0.632
Not relevant	52.4 (66)	0.876	1.12 (0.73–1.74)	0.597
As regards body donation, I believe bureaucracy is^b^				
An obstacle	52.1 (351)		1 (Ref. cat.)	—
Helpful	50.6 (42)		1.09 (0.66–1.81)	0.730
Not relevant	45.5 (121)	0.190	0.85 (0.62–1.17)	0.316
As regards body donation, I believe knowledge and consciousness are^b^				
An obstacle	52.2 (82)		1 (Ref. cat.)	—
Helpful	51.0 (407)		0.90 (0.62–1.31)	0.574
Not relevant	36.8 (25)	0.068	0.58 (0.31–1.09)	0.089

Abbreviations: CI, confidence interval; OR, adjusted odds ratio.

^a^ Possible answers “Slightly/partially agree” or “not at all agree” (categorized as “0”) and “Agree” or “Totally agree” (categorized as “1”).

^b^ 17 missing answers.

^c^ Chi‐squared test.

^d^ Logistic regression model, including 1023 observations, adjusted for all the variables reported in the table.

With regard to the statement “*I consider being a body donor in the future*,” male gender resulted negatively associated (OR: 0.72; 95% CI: 0.55–0.95), whereas age showed no association. Participants who had not attended and did not wish to attend courses involving donated bodies were less likely to consider future donations. Conversely, agreement with viewing body donation as an act of solidarity and a way to interpret the end of life was positively associated with this intention. In addition, perceiving modesty, emotional involvement, and consciousness as non‐obstacles was associated with increased likelihood of considering body donation (Table 8).

**TABLE 8:** Results from the multivariable analysis evaluating the potential predictors of the propensity to body donation.^a^

I consider being a body donor in the future^a^	% (*n*)	*p* ^c^	OR: (95% CI)	*p* ^d^
Overall sample	53.8 (562)	—	—	—
Age in years				
<24	52.0 (331)		1 (Ref. cat.)	—
≥24	56.8 (231)	0.130	1.25 (0.94–1.66)	0.124
Gender				
Female	56.4 (346)		1 (Ref. cat.)	—
Male	50.1 (216)	0.043	0.72 (0.55–0.95)	0.018
In the course of study/faculty where you study/work				
No training activities are organized that involve either observation or dissection of donated bodies / Don't know	57.5 (123)		1 (Ref. cat.)	—
Exercises are organized that involve observation and/or dissection of donated bodies	52.9 (439)	0.230	0.78 (0.56–1.09)	0.146
Have you ever attended courses using donated bodies in institutions different than the one in which you study/work?				
Yes	52.9 (120)		1 (Ref. cat.)	—
No, but I would like to attend a course	55.6 (425)		1.15 (0.83–1.60)	0.407
No, and I would not like to attend a course	32.1 (17)	0.004	0.41 (0.21–0.83)	0.012
Body donation is an act of altruism/solidarity				
No	37.1 (69)		1 (Ref. cat.)	—
Yes	57.5 (493)	<0.001	1.84 (1.28–2.64)	0.001
I agree that body donation is one way of interpreting the end of life				
No/Not known	45.5 (235)		1 (Ref. cat.)	—
Yes	62.1 (327)	<0.001	1.72 (1.30–2.26)	<0.001
As regards body donation, I believe religion/spirituality is^b^				
An obstacle	55.1 (489)		1 (Ref. cat.)	—
Helpful	52.9 (18)		0.92 (0.44–1.90)	0.814
Not relevant	44.5 (45)	0.132	0.59 (0.37–0.95)	0.028
As regards body donation, I believe a sense of modesty for his body is^b^				
An obstacle	50.6 (398)		1 (Ref. cat.)	—
Helpful	66.7 (42)		2.28 (1.24–4.19)	0.008
Not relevant	64.4 (112)	0.001	1.89 (1.30–2.75)	0.001
As regards body donation, I believe emotional participation is^b^				
An obstacle	49.3 (282)		1 (Ref. cat.)	—
Helpful	59.1 (192)		1.37 (1.01–1.85)	0.043
Not relevant	61.9 (78)	0.003	2.07 (1.33–3.21)	0.001
As regards body donation, I believe bureaucracy is^b^				
An obstacle	59.0 (398)		1 (Ref. cat.)	—
Helpful	51.8 (43)		0.65 (0.39–1.07)	0.090
Not relevant	41.7 (111)	<0.001	0.50 (0.36–0.68)	<0.001
As regards body donation, I believe knowledge and consciousness are^b^				
An obstacle	41.4 (65)		1 (Ref. cat.)	—
Helpful	57.5 (459)		1.89 (1.30–2.74)	0.001
Not relevant	41.2 (28)	<0.001	0.98 (0.52–1.85)	0.947

Abbreviations: CI, confidence interval; OR, adjusted odds ratio.

^a^ Possible answers “No” or “I don't know” or “Prefer not to answer” (categorized as “0”) and “Yes” (categorized as “1”).

^b^ 17 missing answers.

^c^ Chi‐squared test.

^d^ Logistic regression model, including 1023 observations, adjusted for all the variables reported in the table.

### Validation

The KMO measure indicated mediocre sampling adequacy (0.64); Bartlett's test rejected the identity hypothesis (*χ* ^2^(6) = 788.7, *p* < 0.001). Cronbach's α for the attitude scale was 0.63, indicating questionable internal consistency. Item‐level MSA values ranged 0.59–0.85. Overall, these figures indicate adequate, though not strong, psychometric properties.

## DISCUSSION

Body donation remains a cornerstone of medical education and research, yet willingness to donate is shaped by factors that extend beyond recognition of its educational value. In this national survey of Italian medical students and residents, willingness to consider body donation was relatively high but coexisted with limited awareness of the legal framework and persistent perceived barriers. Differences between students and residents highlighted how training stage and clinical exposure may influence the weight attributed to knowledge, emotional participation, and cultural or spiritual concerns. Together, these findings support a view of body donation attitudes as multifactorial and dynamic across medical training, with implications for how educational contexts frame and discuss donation (Figure 2).

**FIGURE 2:**
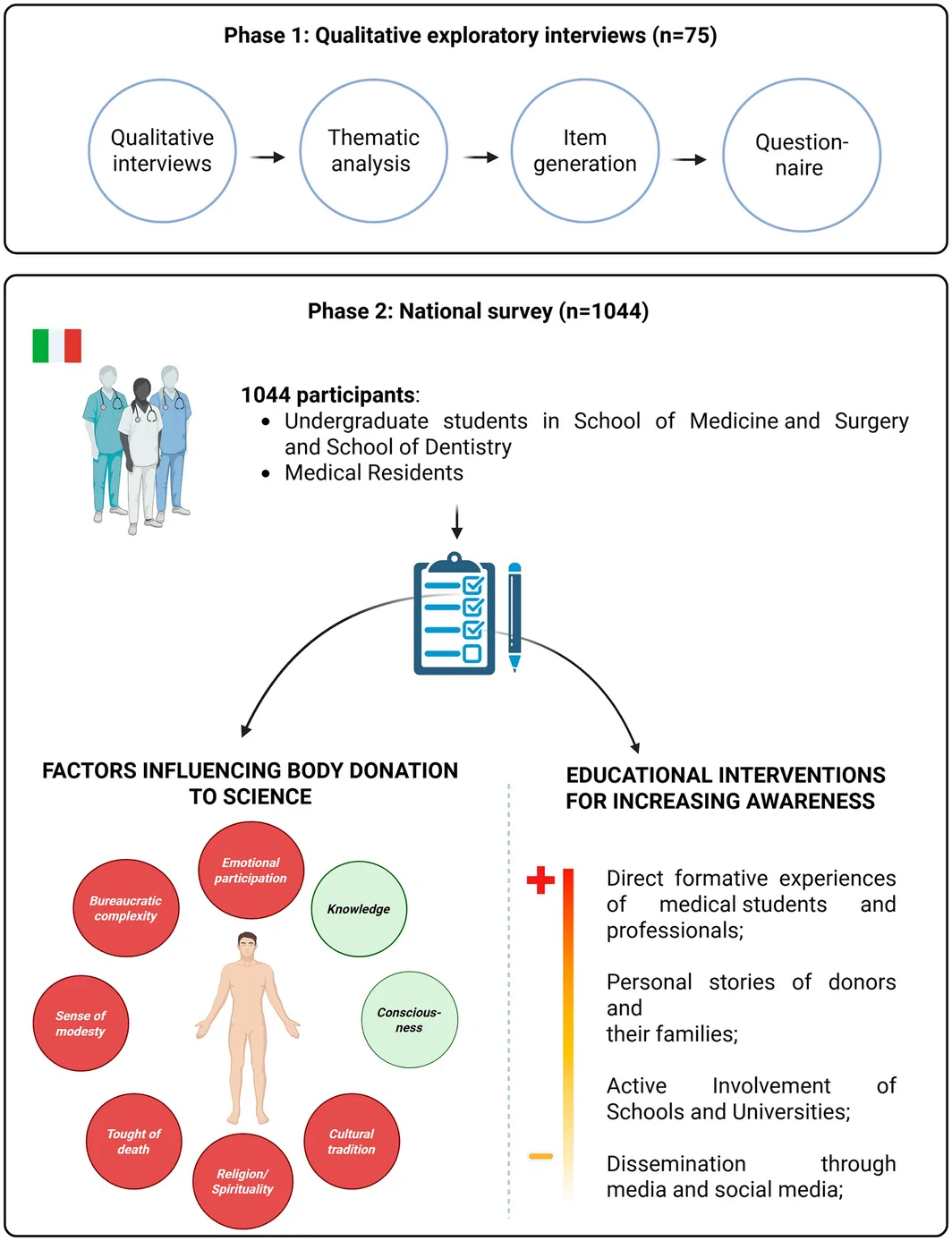
Schematic overview of the study design. The study followed a sequential mixed‐methods approach, in which findings from an initial qualitative phase (75 in‐depth interviews) informed the development of the questionnaire used in the subsequent national survey (*n* = 1044, students *n* = 849, residents *n* = 195). The results highlighted both negative (red) and positive (green) factors influencing decisions about body donation, as well as the strategies considered most effective by participants for increasing public awareness of this choice.

### Educational role of body donation

Training on donated bodies emerges as a fundamental component of medical education. Our findings revealed that most participants had the opportunity to engage with donated bodies, primarily through observational activities, with a smaller portion participating in dissecting activities. Notably, these opportunities were mainly available at the University of Bologna, highlighting the availability of structured body‐based training opportunities compared with other institutions. Beyond university programs, only a minority reported attending extra‐curricular courses on donated bodies at other academic institutions or through private funding. However, many expressed strong interest in pursuing such opportunities, reflecting both the value attributed to hands‐on anatomy education and the need for broader access. Of note, participants with prior exposure to training on donated bodies were more likely to consider donation themselves, further emphasizing the role of such experiences in fostering informed decision‐making and ultimately increasing a person's willingness to donate. In line with this, the findings from the University of Bologna further support the importance of structured educational contexts, as these participants showed higher levels of exposure to donated bodies and greater awareness of body donation compared with those from other institutions. Although these findings should be interpreted with caution, they suggest that the availability of structured educational experiences and institutional commitment to body donation programs may contribute to fostering more informed and reflective attitudes toward donation.

The high level of agreement regarding the educational value of training on donated bodies is consistent with international evidence highlighting the pedagogical role of dissection in consolidating theoretical knowledge and enhancing professional competence.^29^ From an educational perspective, the inclusion of dissection and prosection in the curriculum is not only instrumental in strengthening anatomical knowledge, but also in cultivating respect for human remains and awareness of the ethical dimension of medical training.^6^ Our data further support the integration of extracurricular initiatives, including optional university‐based courses and specialized training activities involving donated bodies, which may ensure broader accessibility and reinforce the role of donated bodies in clinical and surgical education.^30^ Overall, participants strongly highlighted the educational value of this form of training, with almost complete agreement on its importance for acquiring a deep understanding of anatomy and addressing critical situations in clinical practice.

### Awareness and public engagement

Academic programs were frequently reported as the first source of information about body donation among participants. Only a minority became aware of it through alternative sources, including social media, family or friends, and various additional channels. This suggests that universities may represent an important context for initial awareness within this population.^31^ However, despite the significant impact, participants perceived that broader public knowledge remains insufficient, revealing a potential gap between academic education and societal awareness. This perception aligns with previous studies reporting limited public awareness in several countries, where misconceptions and lack of information are recognized as significant barriers to donation.^11^, ^32^

Most respondents indicated awareness campaigns as potentially useful to improve public understanding and engagement. Similar findings have been reported internationally, where educational interventions, storytelling, and community‐based initiatives have proven effective in increasing acceptance of body donation.^8^, ^33^ Participants in our study valued body donation for its contribution to medical education and expressed support for initiatives aimed at increasing public understanding of this practice. This is consistent with evidence showing that educational exposure and positive narratives can enhance acceptance of donation and indirectly benefit healthcare training.^26^, ^34^ Importantly, our analysis underscored participants' preference for strategies based on personal experiences of students and professionals, and stories of donors and their families. This may reflect students' belief that their own narratives and firsthand experiences, given their current and future role as healthcare providers, could be more persuasive in promoting body donation awareness.

Similarly, stories from donors and their families may help address common fears and misconceptions, potentially supporting more informed reflection about body donation. Such approaches, which humanize the concept of body donation, making it more relatable and emotionally resonant, are consistent with previous literature on the role of storytelling in fostering empathy and understanding in healthcare‐related contexts.^35^ For instance, graphic medicine uses comics and graphic narratives to explore healthcare‐related topics.^36^ This approach has emerged as a creative and impactful tool to enhance students' understanding of the topic while addressing the sensitive and often‐taboo nature of death and dissection of donated bodies.^33^ Previous studies have also highlighted its potential to foster empathy, improve communication, and present complex or delicate issues in a more accessible way.^37^, ^38^ Building on this foundation, an interdisciplinary collaboration between the Department of Biomedical and Neuromotor Sciences, the Department of Sociology, and the Department of Design and the Academy of Fine Arts of the University of Bologna led to the publication of the graphic novel “*Il primo paziente. La donazione del corpo in graphic novel*” (Tenuè Publisher) in 2024.^33^, ^39^ Developed as a tool for public engagement, this project employs the comic format to demystify body donation and emphasize its critical role, while fostering a deeper appreciation for this invaluable act. The graphic novel has been presented in exhibitions, academic seminars, and secondary schools to stimulate discussion and reflection on body donation among a wider public. Preliminary informal feedback from these settings suggests that the comic facilitated engagement with the topic and encouraged reflection on its ethical and social dimensions.

These strategies, complemented by the wide reach and effectiveness of digital platforms, offer a promising avenue for awareness campaigns that can effectively engage nearly the entire population. Indeed, as highlighted by Healy^29^, social networks and community‐driven narratives play a pivotal role in shaping attitudes toward donation.^40^ In contexts where family and social influences are particularly strong, decisions around body donation may be shaped by collective opinions rather than individual autonomy, making such community‐focused approaches particularly impactful.^41^, ^42^

### Attitudes toward body donation

Overall, participants expressed favorable attitudes toward body donation, largely perceiving it as an invaluable contribution to education and research, rooted in altruism and generosity. Importantly, the notion of body donation as an inappropriate practice was overwhelmingly rejected, reinforcing the idea that donation is widely regarded as an ethically acceptable and socially valuable act, especially among medical students and professionals.^20^

By contrast, opinions were more divided on whether donation should be interpreted as a way of understanding the end of life, suggesting that cultural, spiritual, and personal factors may shape this perception. For some, body donation may represent a form of continuity and legacy through education and research, whereas for others it may evoke an emotionally difficult engagement with mortality.^20^, ^43^, ^44^

Insights from the qualitative analysis further support this interpretation, highlighting how willingness, reluctance, or uncertainty toward donation reflect different underlying value systems. Altruistic and scientific motivations appear to foster a proactive attitude, while concerns about bodily integrity and emotional discomfort remain significant barriers. Importantly, uncertainty was often linked not to rejection but to informational needs and the involvement of family in decision‐making, emphasizing the relational and socially embedded nature of donation choices. In this sense, attitudes toward body donation emerge not as fixed positions, but as dynamic and potentially modifiable through education, dialogue, and transparent communication.^43^

### Factors influencing willingness to donate

#### Spiritual beliefs and personal values

Several interconnected factors shaped attitudes toward body donation, reflecting the complex interplay between cultural, spiritual, emotional, and personal dimensions. Our data showed that spirituality and cultural aspects often acted as obstacles, with residents reporting a stronger influence than students, possibly due to broader clinical exposure and age‐related experience (Figure 1). It is important to emphasize that spirituality was considered in its broadest sense, encompassing all forms of expression, including non‐religious or not formally recognized. As no denominational data were collected, these findings should be interpreted as reflecting subjective perceptions of spirituality rather than specific doctrinal positions.

However, religious doctrine exerts a profound influence on attitudes toward body donation, with considerable variability arising from differing interpretations of scripture, even within the same religious tradition, as highlighted by Oliver et al.^32^ Dialogue with community and faith leaders has been shown to align donation practices with religious teachings, fostering greater acceptance.^45^, ^46^ For instance, within Christian culture, body donation is generally considered permissible, as the body is believed to no longer contain the soul after death. However, willingness to donate may still vary substantially across cultural and national contexts.^47^ Religious beliefs remain a strong deterrent in some contexts, such as Ghana, even among medical professionals,^48^ whereas in Serbia willingness to donate reflects a complex interaction between religion, ethnicity, and cultural norms, with altruism acting as a potential counterbalancing motivator.^49^

In line with this, participants who considered religion or spirituality irrelevant to body donation were significantly more willing to donate, suggesting that spiritual concerns may act less as fixed barriers and more as interpretative frameworks through which donation is evaluated. This finding underscores the need for culturally sensitive approaches that acknowledge spiritual diversity rather than assuming doctrinal opposition.

#### Emotional and bodily perceptions

Perceptions related to bodily integrity, modesty, and emotional involvement showed heterogeneous effects on willingness to donate. While frequently cited as barriers, these factors were also reported as favoring factors, reflecting heterogeneous and sometimes contrasting cultural perceptions. In this context, “cultural tradition” emerged as a broad construct encompassing shared norms and values surrounding the body and death, including family expectations, respect for bodily integrity, and communal practices of commemoration.

For some individuals, the idea of their body being used post‐mortem may evoke feelings of vulnerability, stemming from cultural, religious, or personal beliefs that emphasize the sanctity and privacy of the human body.^50^ Interestingly, participants who considered modesty irrelevant were more likely to express willingness to donate, suggesting that transparent communication on the respectful handling of bodies could reduce hesitation. Fear of bodily disrespect, often linked to limited exposure to donation procedures, has been widely recognized as a psychological deterrent.^51^, ^52^ In this regard, funeral rituals remain crucial across cultures, as they provide meaning for families and communities and reinforce respect for the deceased.^53^, ^54^, ^55^

#### Administrative and demographic factors

Bureaucratic and procedural aspects were seen as obstacles by most of the participants. Complex regulations, lack of clarity, and extensive paperwork may discourage potential donors, particularly younger individuals who are unfamiliar with such processes. This aligns with broader evidence emphasizing limited awareness of legislation regulating body donation.^21^ Interestingly, participants who viewed bureaucratic aspects as irrelevant to their decision were less likely to consider donation, underlining the importance of addressing both practical barriers and public misperceptions. Gender differences were also observed, with women showing a greater inclination to consider body donation than men. This trend may reflect gender‐related differences in empathy and prosocial behavior, as well as sociocultural expectations that emphasize caregiving roles and altruistic behavior. While such interpretations should be approached cautiously, they highlight the importance of considering demographic factors when designing targeted educational interventions.

#### Educational exposure and training level

Educational exposure emerged as a key determinant shaping emotional responses to body donation. Students, with more limited clinical exposure, tended to view body donation as a learning opportunity, whereas residents, more engaged in patient care, perceived emotional involvement as an obstacle (Figure 1). Similar trends have been described in other studies, showing that increasing professional responsibility and repeated exposure to illness and death reshape emotional processing and ethical sensitivity.^56^ Consistent with this interpretation, participants with direct exposure to body‐based anatomical training were significantly more likely to support body donation, highlighting the value of experiential learning in fostering positive attitudes.^6^, ^29^ In addition, perceptions of death itself differed across the sample.

Moreover, perceptions of death varied across levels of training. While some participants viewed the thought of death as an obstacle, others considered it neutral or as a factor favoring consideration of body donation. Agreement with death as a facilitating factor was more common among older participants and those with prior exposure to anatomical training, suggesting that familiarity with death‐related contexts may promote reflective and altruistic interpretations of body donation. This finding is consistent with the concept of “death literacy,” defined as a set of knowledge and experiences that enable individuals to understand the end‐of‐life and dying care options.^598^ Participants with greater experiential exposure appeared more likely to interpret body donation as a meaningful way of giving continuity to life through education and research. Conversely, younger participants frequently perceived death as abstract or irrelevant to their decision‐making, a pattern that has been described in sociological literature as characteristic of contemporary contexts in which death is distanced from everyday experience.^58^

#### Knowledge and awareness

Finally, knowledge and consciousness emerged predominantly as favorable factors, particularly among students, confirming that educational exposure fosters acceptance (Figure 1). Early exposure through anatomy courses may encourage students to perceive body donation as an essential component of medical education and research.^34^ However, as individuals advance in their careers, the motivating role of knowledge may decline, possibly due to competing influences or the availability of alternative educational tools, such as imaging techniques and virtual dissection.^59^ Nonetheless, awareness alone does not automatically translate into willingness to donate. Even among participants who clearly recognized the educational value of body donation, personal, cultural, and familial reservations often prevail. As Rasania et al. noted, this gap between professional knowledge and personal commitment is significant.^60^ Indeed, our findings suggest that while students and residents recognize the educational value of body donation, personal and familial reservations continue to play a decisive role in individual decision‐making.

### Educational interventions and future directions

In light of the present findings, several implications for educational practice and future research emerged. Although knowledge and awareness were widely recognized as facilitating factors, they were not always sufficient to translate into a personal willingness to donate. This highlights the need for educational approaches that address not only cognitive, but also emotional and value‐based dimensions of decision‐making. Educational interventions may benefit from integrating clear, structured content with reflective and experiential components. Exposure to body‐based training, together with opportunities to engage with personal narratives by students, professionals, donors, and families, may help contextualize body donation within medical education and reduce psychological and cultural barriers identified in this and other studies.

Academic institutions also play a key role in this effort by providing well‐organized learning opportunities that integrate knowledge with practice.^2^ Early exposure during medical training appears particularly relevant, as participants with direct anatomical experience showed more favorable attitudes toward donation. However, to fully understand the societal perception of body donation and to design more effective awareness strategies, it is essential to expand research beyond medical students and professionals. Administering this questionnaire to a broader demographic, including individuals from different age groups, educational backgrounds, and those outside the medical field, could provide valuable insights into the general public's attitudes, concerns, and knowledge gaps regarding body donation.

Ultimately, addressing these barriers requires a multidimensional approach. Public awareness campaigns may combine factual information with narrative‐based approaches, as storytelling and community‐oriented communication have been shown to support engagement with body donation in educational and public settings.^8^, ^33^, ^35^

### Strengths and limitations

This study offers several strengths that enhance its contribution to the understanding of attitudes toward body donation among healthcare professionals and students. First, the large and diverse sample size, comprising 1044 participants, supports the robustness of the quantitative findings. The inclusion of participants across different demographics, including various genders, age groups, and professional backgrounds, allows for a comprehensive analysis of the factors influencing attitudes toward body donation. In addition, the mixed‐methods approach allowed qualitative insights to inform questionnaire development, strengthening the relevance and interpretability of the survey items. The use of inferential statistical methods, such as logistic regression, further strengthens the identification of key predictors associated with willingness to donate.

However, this study is not without limitations. The cross‐sectional design does not allow causal inferences, and attitudes toward body donation may evolve over time or in response to specific educational interventions. The study population consisted predominantly of young individuals undergoing medical training, which limits the generalizability of the findings to the broader population, particularly to older age groups who are more directly involved in end‐of‐life decisions. Hence, conclusions regarding awareness strategies are primarily applicable to educational and training contexts within healthcare. Additionally, the geographical focus on participants primarily from Emilia‐Romagna may limit the generalizability of the findings to other cultural contexts. Attitudes toward body donation are deeply influenced by cultural and religious beliefs, which can vary significantly across different regions and countries.^48^, ^49^, ^61^ ^,^ Data were self‐reported, introducing the possibility of response and social desirability bias, particularly given the sensitive nature of body donation. Finally, although qualitative methods informed the questionnaire design, the survey itself cannot capture the full depth of individual motivations and cultural meanings associated with donation.

## CONCLUSIONS

This study captures the perspectives of Italian medical students and residents, examining factors that are relevant within healthcare training contexts to explore concepts underpinning body donation in this population. While there is widespread recognition of the importance of body donation in advancing medical education and research, significant barriers remain. Although many participants demonstrated positive attitudes and acknowledged the educational value of body donation, cultural and religious beliefs, emotional discomfort, and procedural complexity still influence personal willingness to donate. Importantly, the findings revealed a gap between professional awareness and individual commitment, underscoring that knowledge alone is not sufficient to translate recognition into willingness to donate. Taken together, these results emphasize the central role of academic institutions in shaping informed and reflective attitudes toward body donation, while also highlighting the need to situate educational efforts within broader social and cultural contexts to facilitate awareness of body donation and body donation processes. By clarifying how attitudes evolve during medical training, this study contributes to a better understanding of the challenges and opportunities involved in promoting ethically grounded engagement with body donation in healthcare education.

## AUTHOR CONTRIBUTIONS

**Maria Alessandra Sotgiu:** Writing – review and editing. **Antonietta Fazio:** Writing – original draft; methodology; formal analysis. **Anna Maria Billi:** Writing – review and editing; formal analysis. **Annalisa Plava:** Writing – review and editing; conceptualization; methodology. **Veronica Moretti:** Writing – review and editing; conceptualization; methodology; funding acquisition. **Veronica Macchi:** Writing – review and editing; project administration. **Cecilia Acuti Martellucci:** Writing – review and editing; software; data curation. **Giulia Adalgisa Mariani:** Writing – review and editing; formal analysis. **Simone Lodi:** Writing – review and editing; formal analysis. **Alessandra Ruggeri:** Writing – review and editing; project administration. **Giulio Vara:** Software; formal analysis; writing – original draft; methodology. **Rita Rezzani:** Writing – review and editing; project administration. **Marilisa Quaranta:** Writing – review and editing; formal analysis. **Mario Rende:** Writing – review and editing; project administration. **Valentina Di Felice:** Writing – review and editing; project administration. **Stefano Ratti:** Funding acquisition; resources; project administration; supervision; writing – review and editing; conceptualization. **Andrea Montella:** Writing – review and editing; project administration. **Sergio Castorina:** Writing – review and editing; project administration. **Lucia Manzoli:** Funding acquisition; resources; project administration; writing – review and editing; conceptualization. **Lucio Cocco:** Writing – review and editing; resources. **Guido Carpino:** Writing – review and editing; project administration.

## FUNDING INFORMATION

The research leading to these results has received funding from Alma Idea 2022—Unibo—“Propensioni e ostacoli alla donazione del corpo post‐mortem alla scienza (PRO.DONO)” to Veronica Moretti and Stefano Ratti of the University of Bologna, Italy. Moreover, the presented research received financial support from the Italian Ministry of Health through CENTRO_RIF_DONAZ_SALME_2023 to Lucia Manzoli for the Reference Center for the Preservation and Use of Gifted Bodies of the University of Bologna.

## Supporting information


Data S1.

## Data Availability

The data that support the findings of this study are available on request from the corresponding author. The data are not publicly available due to privacy or ethical restrictions.
