# Supplementary material for: Medical students' perspectives on body donation to science within the Italian context
Source: Anat Sci Educ. 2026 Jul 13;19(10):1649–69. doi: 10.1002/ase.70295 (PMC13654054; doi:10.1002/ase.70295)
Supplement: Supplementary file 1 [file ASE-19-1649-s001.docx]

**Survey: PRO.DONO – Propensioni e ostacoli alla donazione del corpo post-mortem alla scienza**

As part of the **AlmaIdea PRO.DONO** project - Propensioni e ostacoli alla donazione del corpo post-mortem alla scienza - funded by Alma Mater Studiorum (University of Bologna), we invite you to fill out this questionnaire.

It will take about **5 minutes** to answer all questions.

The questionnaire is **ANONYMOUS** and the target audience are **students of the School of Medicine and Surgery and School of Dentistry**, and **medical residents** at national level. Data will be processed in accordance with the provisions of the GDPR and Legislative Decree No. 196/03 and analyzed in aggregate form.

Your participation is essential to probe real knowledge about post-mortem body donation. The results will be essential for planning future awareness-raising activities on a topic of fundamental importance for medical education.

**Section A: Professional training**

**1. In the course of study/faculty where you study/work:**

□ training activities are organized that involve observation of dissected donated bodies (Proceed to question 1.a)

□ training activities are organized that involve dissection of donated bodies (Proceed to question 1.a)

□ training activities are organized that involve both observation and dissection of donated bodies (Proceed to question 1.a)

□ no training activities are organized that involve either observation or dissection of donated bodies (Proceed to question 1.b)

□ don't know (Proceed to question 1.b)

**1.a The training activities are:**

|  | Part of a body | Whole body | Both |
| --- | --- | --- | --- |
| Mandatory |  |  |  |
| Not mandatory |  |  |  |

**1.b Have you ever attended courses using donated bodies in institutions different than the one in which you study/work?**

□ Yes (Proceed to questions 1.c-e)

□ No, but I would like to attend a course (Proceed to question 2)

□ No and I would not like to attend a course (Proceed to question 1.f)

**1.c In which city/country?**

**______________________**

**1.d How was the course you chose funded?**

□ Privately

□ Scholarship

□ Agreement/Partnership

□ Sponsored

□ Other: _____________

**1.e How would you describe your experience in one word?**

__________________________

**1.f Why would you not participate?**

**______________________________**

**2. How much do you agree with the following statement: "Training on donated bodies is essential for gaining a deep understanding of anatomy and addressing potential critical situations/emergencies in the healthcare field more effectively."**

1	2	3	4	5

**Section B: The donation**

**3. Are you aware of the possibility of donating your post-mortem body to science?**

□ Yes (Proceed to question 3.b)

□ No (Proceed to question 3.a)

**3.a. What is post-mortem body donation to science?**

Post-mortem body donation refers to the temporary use of one's body for scientific research and educational purposes by an institution, for a defined period, after which the body is returned to the family.

On March 19, 2020, the law No. 10/2020, titled " Rules regarding the disposition of one's body and post-mortem tissues for study, training, and scientific research purposes" came into force. This law established the procedures to formally express the willingness to donate own’s body for scientific and educational purposes.

The Anatomy Center of the University of Bologna, Alma Mater Studiorum, has been recognized as National Reference Center for the Preservation and Use of Gifted Bodies by the Italian Ministry of Health.

**3.b How did you first learn about the possibility of donating your post-mortem body to science?**

□ Colleagues

□ Course of study attended

□ Friends

□ Family/relatives

□ Scientific literature

□ Media, social media

□ Conferences, seminars, meetings

□ Other: ___________

**4. How well do you think the topic of post-mortem body donation to science is known?**

□ Not at all

□ Slightly

□ Moderately

□ Very well

**5. Do you think an awareness campaign on this topic could be useful/necessary?**

□ Yes

□ No

□ I don’t know

**6. Do you think organ donation is compatible with post-mortem body donation to science?**

□ Yes

□ No

□ I don’t know

**7. Are you aware of the law No. 10/2020, which regulates post-mortem body donation to science?**

□ Yes (Proceed to question 7.b)

□ No (Proceed to question 7.a)

**7.a What is post-mortem body donation to science and how is it regulated?**

Post-mortem body donation refers to the temporary use of one's body for scientific research and educational purposes by an institution, for a defined period, after which the body is returned to the family.

On March 19, 2020, the law No. 10/2020, titled " Rules regarding the disposition of one's body and post-mortem tissues for study, training, and scientific research purposes" came into force. This law established the procedures to formally express the willingness to donate own’s body for scientific and educational purposes.

The Anatomy Center of the University of Bologna, Alma Mater Studiorum, has been recognized as National Reference Center for the Preservation and Use of Gifted Bodies by the Italian Ministry of Health.

**7.b. We ask you to think about the act of donating your post-mortem body to science. For each statement, please indicate your level of agreement.**

|  | Strongly disagree | Disagree | Agree | Strongly agree |
| --- | --- | --- | --- | --- |
| Body donation is an act of altruism/solidarity |  |  |  |  |
| Body donation is useful to healthcare progression through training and research |  |  |  |  |
| Body donation is a way of interpreting the end of life |  |  |  |  |
| Body donation is an inappropriate act |  |  |  |  |

**8. Do you know anyone who has expressed consent for post-mortem body donation to science?**

□ Yes

□ No

**9. Would you consider becoming a body donor in the future?**

□ Yes

□ No

□ I don’t know

□ I prefer not to answer

**10. Can you explain your previous answer?**

_____________________________________________

**Section C: Awareness**

**11.What personal considerations do you think may influence post-mortem body donation to science? Please indicate the option you find appropriate for each factor.**

|  | Obstacle | Predisposition | Not relevant |
| --- | --- | --- | --- |
| Religion / spirituality |  |  |  |
| Thoughts about death |  |  |  |
| Sense of modesty about one's body |  |  |  |
| Emotional partecipation |  |  |  |
| Bureaucratic process |  |  |  |
| Knowledge and awareness |  |  |  |
| Cultural tradition |  |  |  |

**12. Choose the method you think would be most useful for raising awareness about post-mortem body donation to science:**

□ Sharing the stories of donors and their families

□ Sharing the training experiences of students and professionals

□ Creating specific associations

□ Providing information through media and social media

□ Providing information through Schools and Universities

□ Providing information through specific healthcare facilities (e.g., blood donor centers)

□ Receiving information through the General Practitioner

□ Other: ______________________

**Section D: Socio-demographic profile**

**13.Gender:**

□ Female (F)

□ Male (M)

□ Other: __________

**14. Year of Birth:**

_________________

**15. Nationality:**

□ Italian

□ Other: __________

**16. Region where you study/work:**

_________________

**17. University Affiliation:**

_________________

**18. Course of Study:**

□ Medicine and Surgery

□ School of Dentistry

□ Other: __________

**19. Current Position:**

□ Student of Medicine and Surgery or School of Dentistry

□ Medical resident

□ Other: __________
